# Lifespan extension without fertility reduction following dietary addition of the autophagy activator Torin1 in *Drosophila melanogaster*

**DOI:** 10.1371/journal.pone.0190105

**Published:** 2018-01-12

**Authors:** Janet S. Mason, Tom Wileman, Tracey Chapman

**Affiliations:** 1 School of Biological Sciences, University of East Anglia, Norwich Research Park, Norwich, United Kingdom; 2 Norwich Medical School, University of East Anglia, Norwich Research Park, Norwich, United Kingdom; Inha University, REPUBLIC OF KOREA

## Abstract

Autophagy is a highly conserved mechanism for cellular repair that becomes progressively down-regulated during normal ageing. Hence, manipulations that activate autophagy could increase lifespan. Previous reports show that manipulations to the autophagy pathway can result in longevity extension in yeast, flies, worms and mammals. Under standard nutrition, autophagy is inhibited by the nutrient sensing kinase Target of Rapamycin (TOR). Therefore, manipulations of TOR that increase autophagy may offer a mechanism for extending lifespan. Ideally, such manipulations should be specific and minimise off-target effects, and it is important to discover additional methods for ‘clean’ lifespan manipulation. Here we report an initial study into the effect of up-regulating autophagy on lifespan and fertility in *Drosophila melanogaster* by dietary addition of Torin1. Activation of autophagy using this selective TOR inhibitor was associated with significantly increased lifespan in both sexes. Torin1 induced a dose-dependent increase in lifespan in once-mated females. There was no evidence of a trade-off between longevity and fecundity or fertility. Torin1-fed females exhibited significantly elevated fecundity, but also elevated egg infertility, resulting in no net change in overall fertility. This supports the idea that lifespan can be extended without trade-offs in fertility and suggest that Torin1 may be a useful tool with which to pursue anti-ageing research.

## Introduction

Many diseases show a distinct increase in incidence or severity with age. Hence, it is of paramount importance to understand the molecular and cellular mechanisms that regulate ageing in order to minimise health impacts in an ever-older human population. Research over the last two decades reveals that several linked and highly conserved repair, growth and nutrient sensing pathways (autophagy, ‘target of rapamycin’ (TOR) and insulin and insulin-like growth factor (IGF) signaling (IIS)) are intimately involved in determining length of life (e.g. [1–5]). In this study we focused on the links between TOR and autophagy in lifespan determination through specific manipulations of the TOR pathway.

Down regulation of the protein kinase TOR is reported to increase lifespan [3,4]. TOR is highly conserved across eukaryotes and controls several fundamental cellular functions including autophagy—an important and highly conserved cellular repair mechanism [6,7]. TOR is a major regulator of cellular growth and proliferation [3,8–11] and is comprised of two differentially regulated protein complexes TOR complex 1 (TORC1) and TOR complex 2 (TORC2). TORC1 and 2 have distinct substrate specificities and are differentially sensitive to the TOR inhibitor rapamycin [8,9]. TORC1 promotes anabolism and inhibits catabolism by blocking autophagy through the phosphorylation of the *ULK1-Atg13-FIP200* complex [12]. TORC2 is known to be insensitive to rapamycin. It’s role in proteins synthesis isn’t yet clear [10], though it plays roles in many cellular processes via the AGC kinases and is implicated in keratinocyte survival and cancer development [13].

The effects of TOR on autophagy are of interest in the context of ageing [14]. It is known for example, that autophagy is naturally down-regulated as a result of normal ageing [15]. The function of autophagy is to repair cellular damage, leading to the suggestion that manipulations that activate autophagy might increase lifespan by maintaining damage surveillance and increasing cellular repair. Consistent with this, over-expression of specific autophagy genes has been shown to extend lifespan in yeast, flies and human cells [2]. In general, manipulations involving changes to autophagy or autophagy genes are increasingly being reported to be associated with lifespan (e.g. [14,16–19]). Linking the two processes, it has been shown that the specific inhibition of TOR, which in turn activates autophagy, results in extension of lifespan in various species [20,21]. The TOR pathway can be inhibited, and hence autophagy activated, by inactivating TORC1 through treatment of cells with rapamycin or via nitrogen starvation [5,22]. This increase in lifespan due to inhibition of TOR could potentially be via TOR’s effects on protein synthesis. However, research on *C*. *elegans* suggests a more direct role of autophagy in the modulation of longevity, because inactivating autophagy genes specifically prevents the inhibition of TOR activity from extending lifespan. This finding suggests that the TOR pathway and autophagy act via the same signalling pathway to influence lifespan [3]. However, it should also be noted that inhibition of TOR leads to decreased translation as well as increased autophagy [14], hence it can be important to distinguish whether either or both pathways are most associated with lifespan effects.

Existing pharmacological agents can be used to regulate TOR and influence lifespan, as described above. However, some reagents can have low effectiveness and/or off target effects. This prompts a need for further investigation of the nature and importance of any secondary effects of the TOR and autophagy regulators employed as well as the search for additional, and potentially cleaner, methods of manipulating these pathways. In this study, we investigated one such additional method—the effect of dietary addition of Torin1 on lifespan in the *Drosophila melanogaster* model system.

Torin1 is a well-established activator of autophagy via inhibition of the TOR pathway [23,24]. Guertin and Sabatini [23] reported the synthesis of Torin1, which inhibits TOR with a higher degree of selectivity than other previously used pharmacological activators, e.g. rapamycin [23–25]. Part of the mechanism of action of Torin1 is reported to be to suppress the rapamycin-resistant functions of TORC1 that are necessary to reduce autophagy [24]. In addition, unlike rapamycin, Torin1 is reported to inhibit kinase function in both TORC1 and TORC2 complexes [24] potentially giving it greater effectiveness, as it is a dual mTOR inhibitor [26,27]. Torin1 inhibits cell growth and proliferation to a much greater degree than rapamycin and may represent a more effective and specific inhibitor than either spermidine or rapamycin [2,26,27].

The main interest to date in pharmacological interventions affecting the TOR and autophagy pathways has been to investigate lifespan extension. However, it is important that any associated effects on reproduction are also examined. Increased lifespan but decreased reproductive function would not represent a positive outcome for lifespan interventions with therapeutic potential. Such trade-offs commonly occur (e.g. [5,28]) but are often not measured or reported in lifespan studies. Although reduced fecundity might often trade-off with extended lifespan, there is often no simple correlation of fecundity with longevity [29,30]. As an example, extended lifespan is also observed in sterile, non egg-laying females treated with rapamycin, hence lifespan extension due to rapamycin treatment is not causally linked to egg production *per se* [5,31]. In this study we initiated an investigation into the effects on lifespan and reproductive success of Torin1 supplied via the diet, in once-mated and continually mated *D*. *melanogaster* females, and on the lifespan of once-mated males.

## Materials and methods

### Determination of autophagy activation by dietary administration of Torin1

We used Western Blotting to confirm the actvation of autophagy via cleavage of the *Atg8* gene protein into Atg8-I and Atg8-II [32] following dietary administration of Torin1, (S1 Methods, S1 and S2 Figs).

### Dietary delivery of Torin1

We used a design in which flies were fed on live yeast paste plus carrier control or Torin1, placed onto a standard agar base. We used this method to ensure that differences in lifespan were due to Torin1 treatment and to minimise any ancillary differences in dietary intake. This method was chosen on the basis of pilot tests of different food delivery methods as that which most effectively allowed females to feed on medium containing Torin1 (where the only food available contained Torin1), while matching female fecundity to that of females maintained on a normal diet with added live yeast (S1 Table). Torin1 is not water soluble, and the carrier used was dimethyl sulfoxide (DMSO). In additional pilot experiments we tested the effect of DMSO per se on lifespan in this experimental set up and found no significant effect of DMSO on once mated female lifespan at the dose used in these experiments (S3 Fig). However, we note that the lifespans we generally observed in our experiments are shorter than would be the expected for individuals held on normal culture media in the absence of live yeast and DMSO.

### Effect of Torin1 dose (0–10μM) on once-mated female survival, fecundity and fertility

Fly rearing was carried out in a humidified 25°C controlled temperature room with a 12/12 light-dark cycle using the wild-type Dahomey strain of flies. Larvae were raised at a density of 100 per vial to standardise density and reduce environmentally-determined differences in body size. SYA food medium (970 mL distilled water, 15 g agar, 50 g sugar, 100 g brewer’s yeast, 30 mL Nipagin solution (10% w/v solution in ethanol), 3 mL propionic acid) was used for rearing the experimental flies.

Females were separated from males using CO_2_ three days after eclosion. This ensured that at least one mating had occurred and that all individuals could therefore be considered ‘once-mated’. Single, once-mated females were then allocated at random to one of five food treatment groups in replicates of *n* = 40 each. The treatments were carrier only (DMSO) control, 0.5 μM, 1.0 μM, 5 μM and 10 μM Torin1. Each food vial comprised 7 mL of agar with a drop of live yeast paste containing one of the Torin1 treatment doses or carrier control. Females were given a new food vial every 24 hours monday to friday until egg production ceased, and then three times a week until death.

The live yeast paste containing Torin1 (Tocris Bioscience, Catalogue Number 4247) or dimethyl sulfoxide (DMSO) carrier control was made up by adding 5 μL of the required dilution of Torin1 or DMSO carrier to 10 mL distilled water, and then adding 6 g live yeast granules. The paste was deposited as a droplet (2 mm diameter) on the agar in the bottom of each vial. This design ensured treatments were consistent and any differences in lifespan were likely to be due to the addition of Torin1 to the diet. Flies were transferred during the experiment between vials without using anaesthesia.

Female longevity was recorded daily until all females were dead. Individuals that became stuck in the food, escaped or were accidentally killed were entered as censors in the data analysis. Eggs laid in the food vials over 24 hours were counted 2 times each week. The vials were then incubated and the number of unhatched eggs counted the following day to determine egg hatchability. Egg counts were ceased when half the remaining females in a treatment stopped laying eggs or where there were fewer than 5 females remaining in a treatment. The total number of eggs produced over the lifetime of each female in each treatment was summed separately for the total, hatched and unhatched numbers of eggs.

### Effect of 1 μM Torin1 on once-mated and continually mated female survival and fertility and on once-mated male survival

Fly rearing and handling was carried out as in the experiment above. Females and males were collected from mixed sex cultures three days following eclosion, using CO_2_, and were therefore considered ‘once-mated’. Females and males were randomly allocated to one of the 6 treatment groups (*n* = 40 for each). For this experiment a single dose of Torin1 (1 μM) was used. The treatments were: once-mated females, control versus Torin1 diet; once-mated males, control versus Torin1 diet and continually mated females, control versus Torin1 diet. In the continually-mated females, males were replaced with fresh 4 day-old males every 7 days to ensure reproductive activity remained high. To control for the CO_2_ exposure all females and males in the once-mated treatment groups were also similarly CO_2_ anaesthetized each week. Flies were tipped onto new food daily until week 4 and then three times a week until death.

Longevity was recorded daily. As above individuals that became stuck in the food, escaped or were accidentally killed were entered as censors. Eggs laid in the food vials over 24 hours were counted 2 times each week. The vials were then incubated and the number of unhatched eggs counted the following day to determine egg hatchability. For the once-mated and continually exposed females total egg productivity for each individual over their lifetimes (total, hatched and unhatched eggs) was determined. For the once-mated male experiment only survival was recorded.

### Statistical analysis

Statistical analysis was performed using SPSS v 18 [33] and R [34]. Survival data were analysed using Log Rank tests or Cox Regression analyses [33]. Fecundity and fertility data were analysed using ANCOVA to test for the treatments differences over time while accounting for within-subjects repeated measures [34]. The R glm package with a Poisson error distribution was used to account for the error structure of the data. Model simplification via Analysis of Deviance was used to generate, where possible, simplified models of the patterns of fecundity and fertility variation. Fecundity and fertility data were tested for age specific differences in more detail using ANOVA or Kruskal Wallis tests, depending on whether the data were normally distributed. Age specific fecundity and fertility data were tested for normality using Kolmogorov-Smirnov tests and for homogeneity of variance using Levene’s tests. Tests were conducted on the data for each day and corrected for the number of tests using the sequential Bonferroni procedure [35].

## Results

### Determination of autophagy activation by Torin1

Western blotting confirmed that the administration of Torin1 in the diet activated autophagy above control diet levels at all doses of Torin1 in the once-mated females from the first experiment (S1 Fig). Increasing doses of Torin1 up to 5 μm successively increased the level of autophagy activation. Autophagy activation then slightly decreased at the highest level of 10 μm Torin1. Dietary administration of Torin1 also activated autophagy above control diet levels in once-mated females and males and in continually mated females all fed 1 μM Torin1 in the second experiment (S2 Fig). In once-mated and continually mated females, autophagy activation following 5 days on 1 μM Torin1 diets was higher than in individuals maintained on the control diets, but was not as high as for the starvation diet females maintained on agar only food. In once-mated males, autophagy activation on the 1 μM Torin diet exceeded that of males maintained on the starvation agar only diet. Together, the results provided evidence that autophagy was consistently activated by Torin1 in the diet in both experiments.

### Effect of Torin1 (0–10μM) on once-mated female survival, fecundity and fertility

#### Survival

**Fig 1:**
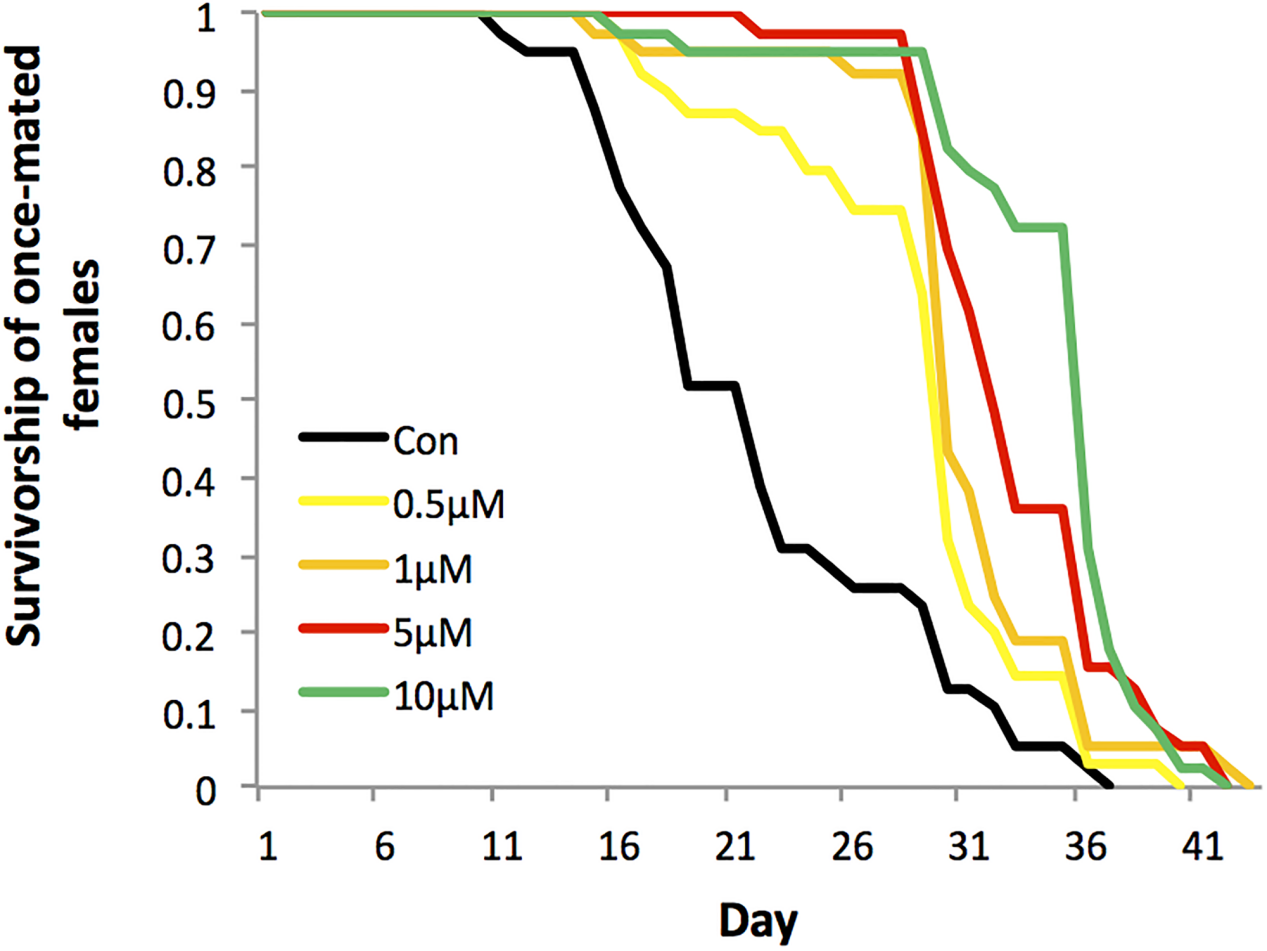
Effect of Torin1 on survivorship of once-mated females. Females were exposed to dimethyl sulfoxide (DMSO) carrier control, 0.5 μM, 1 μM, 5 μM or 10 μM Torin1 in the diet. There was a significant extension in once-mated female longevity observed with increasing doses of Torin1 (Log Rank *X*^*2*^ = 52.106, *df* = 1, *p* < 0.001).

There was a significant increase in lifespan in once-mated Torin1 fed females and evidence for a consistent dose response, with increasing Torin1 leading to ever-greater lifespan. A significant extension in once-mated female longevity was observed with increasing doses of Torin1 in the diet (Log Rank *X*^*2*^ = 52.106, *df* = 1, *p* < 0.001, Fig 1). Females held on the carrier (dimethyl sulfoxide, DMSO) control diet had a mean lifespan of 22 days. This increased with the amount of Torin1 added to the diet to a mean of 28 days (0.5 μM Torin1), 30 days (1 μM Torin1), 33 days (5 μM Torin1) and 35 days (10 μM Torin1). Hence the greatest extension of lifespan (of 13 days, ~60% extension over the control) was observed between the control females and those held on the 10 μM Torin1 diet.

#### Age-specific fecundity and fertility

**Fig 2:**
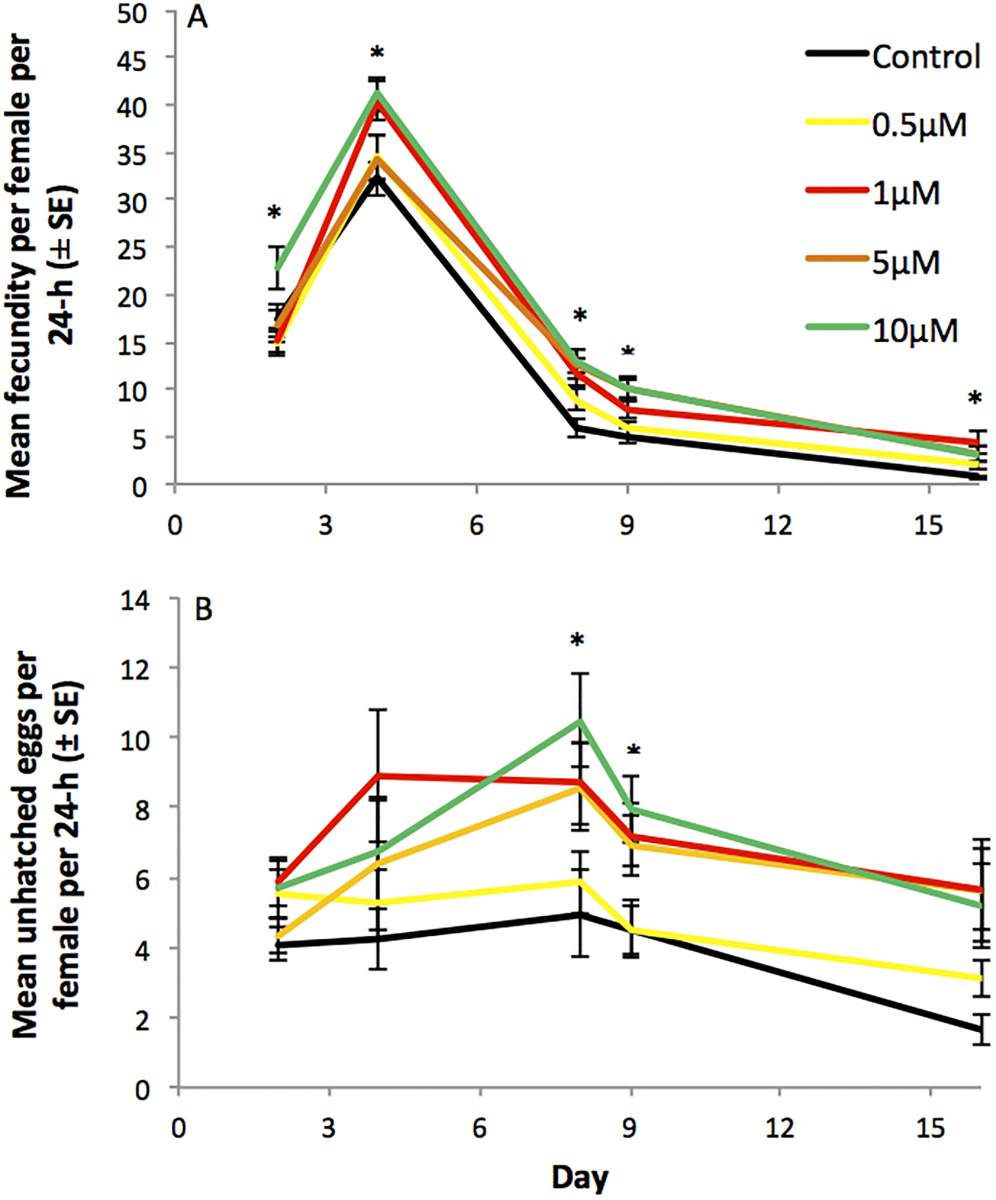
Effect of Torin1 on age-specific fecundity of once-mated females shown in Fig 1. (A) Fecundity (mean eggs per female per 24-h ± SE) of once-mated females exposed to DMSO carrier control, 0.5 μM, 1 μM, 5 μM or 10 μM Torin1 in the diet. (B) Number of unhatched eggs (mean per female per 24-h ± SE) of once-mated females exposed to DMSO carrier control, 0.5 μM, 1 μM, 5 μM or 10 μM Torin1 in the diet. Asterisks indicate a significant difference between groups (ANOVA, *p* < 0.05).

Age-specific fecundity data were analysed using analysis of covariance (ANCOVA). This was followed by analysis of variance (ANOVA) to test for differences in fecundity on individual days, corrected for multiple comparisons using a sequential Bonferroni procedure (Fig 2A). There were significant increases in fecundity between groups over time (*P* = 0.001) and a significant effect of diet treatment (*p* = 0.05). In the age-specific fecundity analyses, significant differences in fecundity were seen on all days tested, (Fig 2A). Post hoc Tukey tests showed that on day 2 the females fed 10 μM Torin1 laid significantly more eggs than females fed 0.5 and 1 μM Torin1 (*p* = 0.011). On subsequent days, control females laid significantly fewer eggs than flies fed 10 μM Torin1 (*p* = 0.027, day 4), 1, 5 and 10 μM (*p* ≤ 0.016, day 8) or 5 μM Torin1 (*p* = 0.036, day 16). On day 9 both the control and the flies fed 0.5 μM Torin1 laid significantly fewer eggs than those fed 5 and 10 μM Torin1 (*p* ≤ 0.022). There was therefore a pattern of Torin1 fed females exhibiting higher age-specific fecundity. Analyses of age-specific fertility data revealed a tendency for higher egg infertility in the females fed Torin1 (Fig 2B). ANCOVA revealed a significant interaction between food and time for egg infertility (*p* = 0.05). Age specific analyses showed significant differences in egg infertility on day 8 and 9 (*p* < 0.05 following Bonferroni correction). On day 8 control females laid significantly fewer unhatched eggs than females held on the 5 and 10 μM Torin1 diets and on day 9 the flies fed 0.5 μM Torin1 laid significantly fewer unhatched eggs than those fed 10 μM Torin1.

#### Lifetime egg productivity

**Fig 3:**
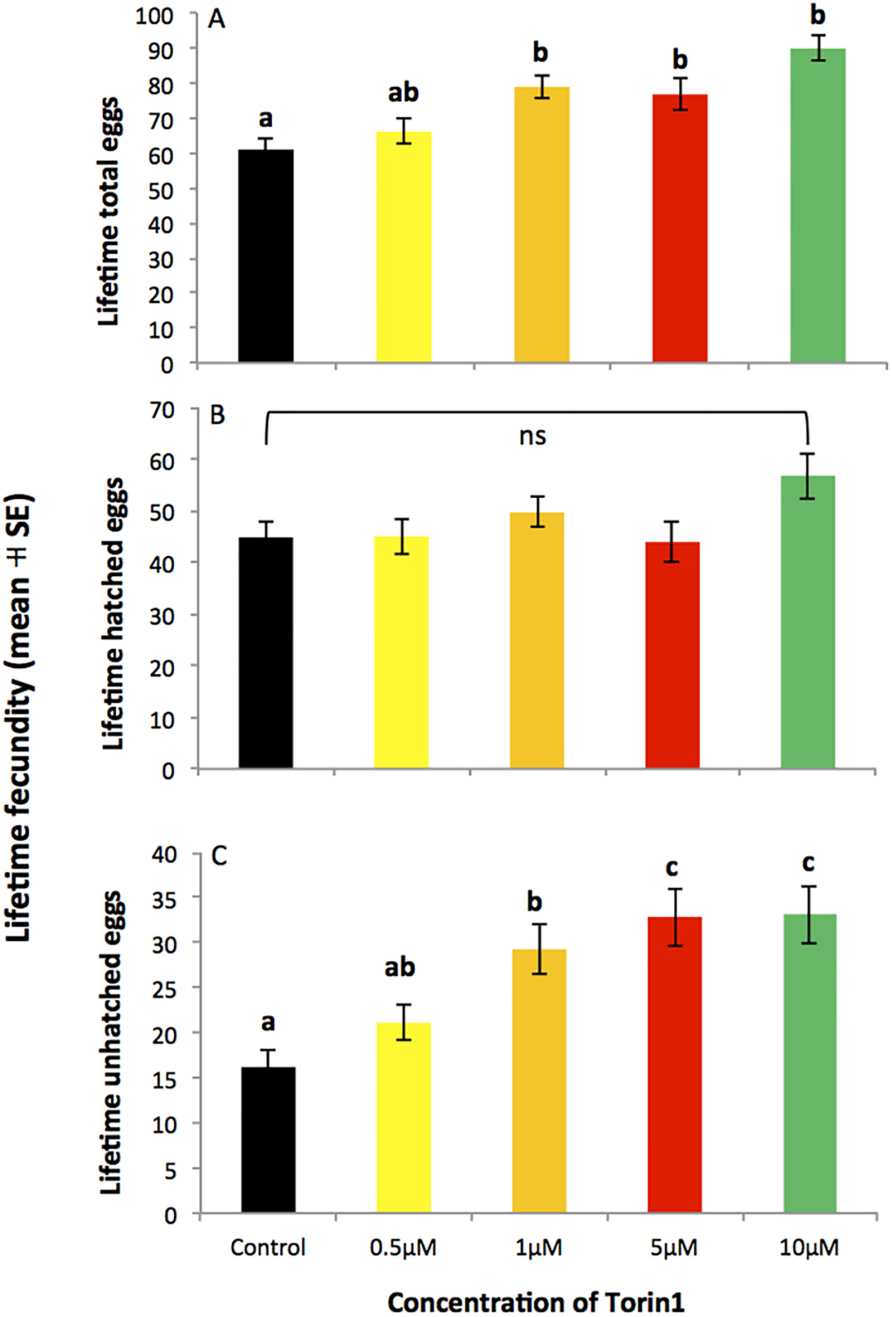
Effect of Torin1 on lifetime fecundity of once-mated females shown in Fig 1. Lifetime egg production (mean ± SE) of once-mated females exposed to DMSO carrier control, 0.5 μM, 1 μM, 5 μM or 10 μM Torin1. (A) total lifetime fecundity, (B) lifetime number of hatched eggs, (C) lifetime number of unhatched eggs. Letters not in common indicate a significant difference between groups (Tukey post hoc test, *p* < 0.05). ns = not significantly different (*p* > 0.05).

Lifetime fecundity and fertility data were consistent with the age-specific analysis described above, and revealed that Torin1 increased total lifetime fecundity but not lifetime fertility (Fig 3A–3C). A significant increase was seen in lifetime fecundity (*F*_4,195_ = 9.430, *p* < 0.001, Fig 3A) and in lifetime unhatched eggs (*F*_4,195_ = 7.784, *p* < 0.001, Fig 3C) as the Torin1 dose increased. The integration of these two effects however resulted in no net difference in the number of lifetime hatched eggs between the different treatments (*F*_4,195_ = 2.220, *p* = 0.068, Fig 3B).

### Effect of 1 μM Torin1 on once-mated and continually mated female survival and fertility and on once-mated male survival

#### Survival

**Fig 4:**
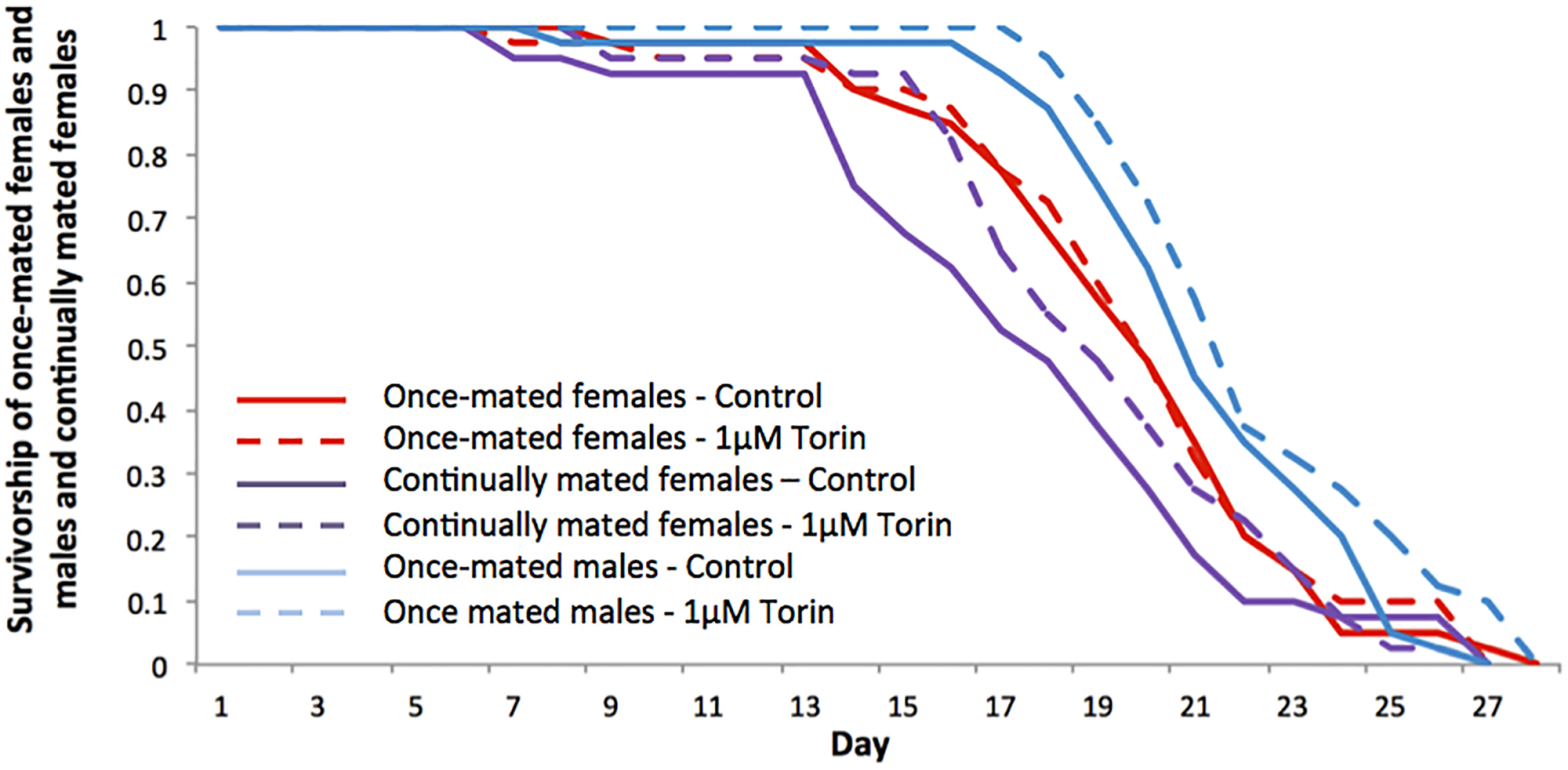
Effect of Torin1 on survivorship of once-mated females, continually mated females and once-mated males. Flies were maintained on DMSO carrier control or 1 μM Torin1 diets. There were significant differences in survival between once-mated females, continually mated females and once-mated males maintained as adults on 1 μM Torin1 versus control medium (*F*_5,234_ = 7.032, *p* < 0.001, Fig 4). There was a significant overall lifespan extension following 1 μM Torin1 treatment in comparison to controls (F_1,234_ = 4.075, *p* = 0.045) due to lifespan extension in continually-mated females and in once-mated males. Once-mated females lived longer than continually mated (*F*_1,234_ = 5.134, *p* = 0.024) and there was a significant effect of sex, with males living significantly longer than females (*F*_1,234_ = 10.517, *p* = 0.001).

There were significant differences in survival between once-mated females, continually mated females and once-mated males maintained as adults on 1 μM Torin1 versus control medium (*F*_5,234_ = 7.032, *p* < 0.001, Fig 4). There was a significant overall lifespan extension following 1 μM Torin1 treatment in comparison to controls (F_1,234_ = 4.075, *p* = 0.045). This effect derived from lifespan extension in continually-mated females and in once-mated males, but not in the once-mated females as observed in the first experiment. There was also a significant effect of mating status, with once-mated flies living longer than continually mated females (*F*_1,234_ = 5.134, *p* = 0.024). A significant effect of sex was also seen, with males living significantly longer than females (*F*_1,234_ = 10.517, *p* = 0.001).

#### Age-specific fecundity and fertility

**Fig 5:**
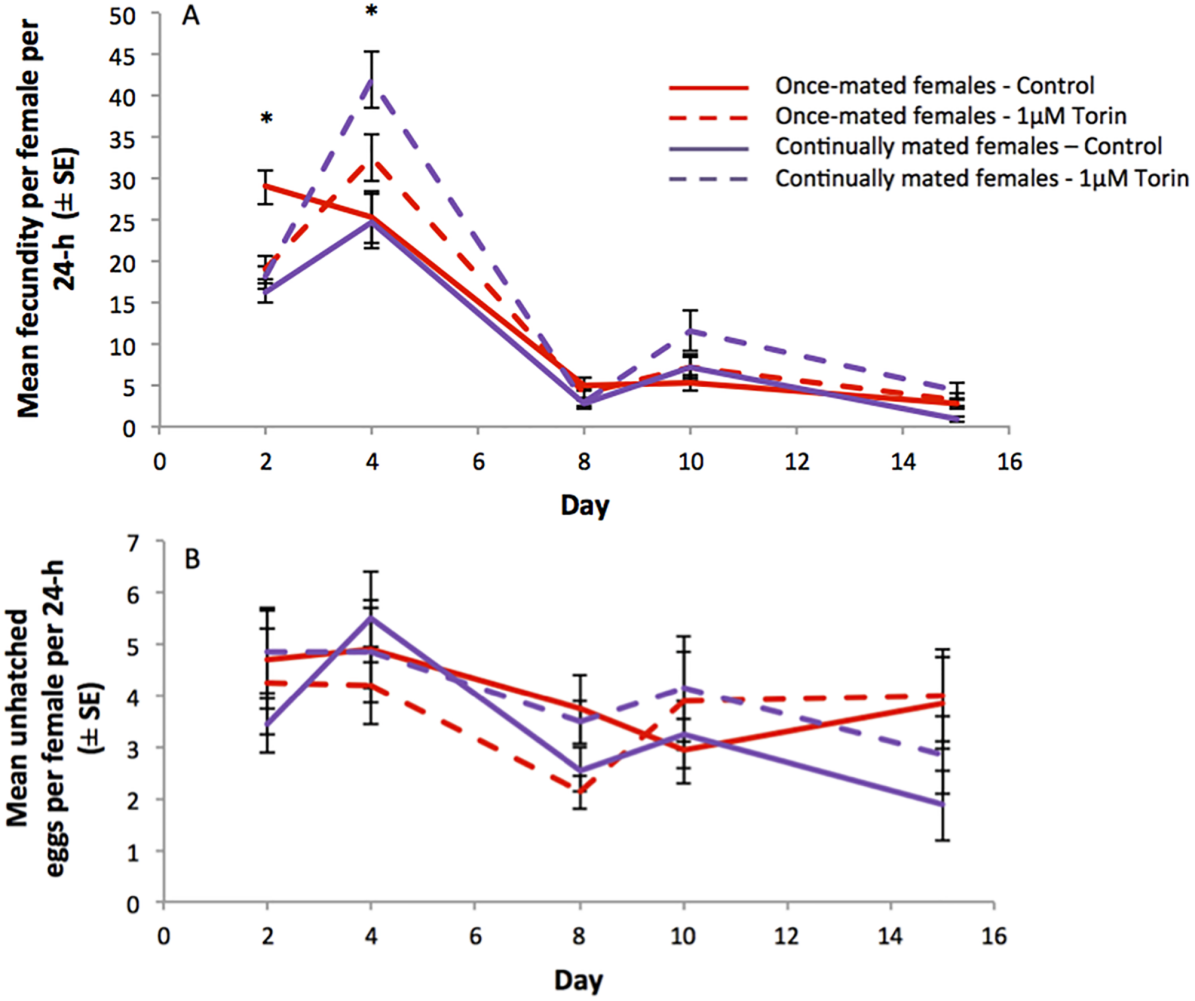
Effect of Torin1 on age-specific fecundity of the once-mated and continually mated females shown in Fig 4. (A) Fecundity (mean eggs per female per 24-h ± SE) of once-mated and continually mated females maintained on DMSO carrier Control or 1 μM Torin1 diet. (B) Egg fertility (mean unhatched eggs per female per 24-h ± SE) of once-mated and continually mated females maintained on DMSO carrier control or 1 μM Torin1 diet. Asterisks indicate a significant difference between groups (ANOVA, *p* < 0.05).

There was a significant difference in the pattern of age-specific fecundity over time for once-mated versus continually mated females (ANCOVA, *p* = 0.001, Fig 5A). This was evident as a significant interaction between mating status and time (*p* = 0.01) and between food type and time (*p* = 0.05). To determine with more precision where these differences lay, analyses of age-specific fecundity, corrected for multiple comparisons were again conducted as described above. These analyses revealed significant differences in fecundity on day 2 and 4 (*p* < 0.05). On day 2 there was a significant interaction between mating status and diet (*p* = 0.001), with higher fecundity in the once-mated control females compared to all other groups (*p* < 0.001). The significant main effect of diet did not map onto consistent differences across the once- or continually mated females (*p* = 0.014). On day 4, consistent with the results from the first experiment, there was a significant increase in fecundity in once- and continually mated females held on the Torin1 diets compared to the controls (*p* < 0.001). ANCOVA analysis of age-specific egg infertility (Fig 5B) revealed no significant differences between the different Torin1 treatments or between female mating status treatments.

#### Lifetime egg productivity and fertility

**Fig 6:**
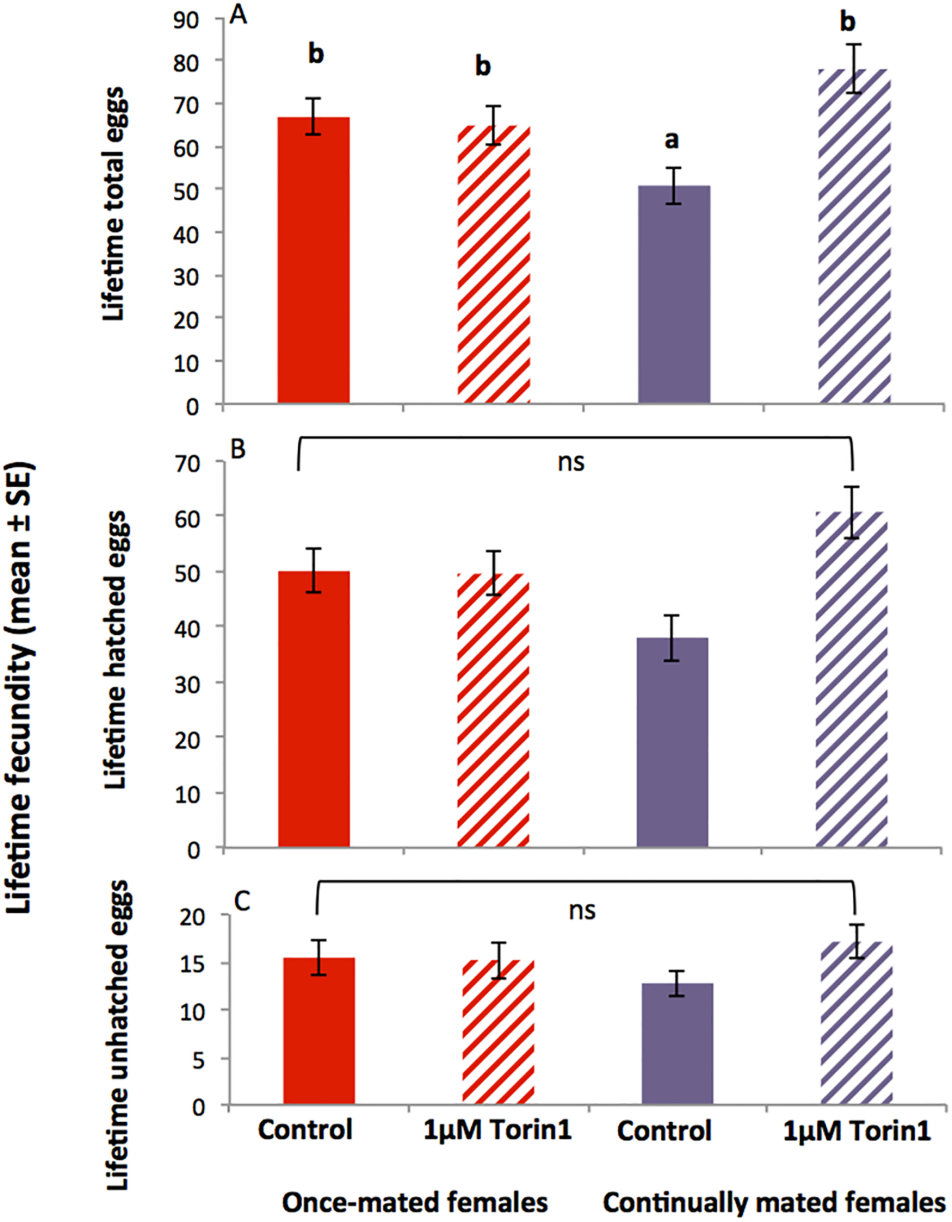
Effect of Torin1 on lifetime egg production in the once-mated and continually mated females shown in Fig 4. Lifetime egg production (mean ± SE) of once-mated females and continually mated females maintained on control or 1μM Torin1 diet. (A) total lifetime fecundity, (B) lifetime number of hatched eggs, (C) lifetime number of unhatched eggs. Letters not in common indicate a significant difference between groups (Tukey post hoc test, *p* < 0.05). ns = not significantly different (*p* > 0.05).

There was a significant interaction between diet and mating status (F_1,156_ = 9.572, *p* = 0.002) as well as a significant main effect of diet itself on lifetime total fecundity (F_1,156_ = 7.150, *p* = 0.008, Fig 6A). Post-hoc testing showed that continually mated, but not once-mated, Torin1 fed females produced significantly more eggs in their lifetimes than did controls (*p* < 0.001). There were no significant differences in the numbers of hatched or unhatched eggs for either mating regime (Fig 6B and 6C).

## Discussion

The main finding was that the addition of Torin1 to the diet activated autophagy and led to significant lifespan extension in both sexes. Significant lifespan extension was observed in once-mated females and males, and females that were held continuously with males. A significant dose response was observed in once-mated females, where longevity increased significantly with increasing concentrations of Torin1 added to the diet (Fig 1). Elevated egg production was observed in females fed Torin1, but overall this did not result in higher overall fertility, owing to higher egg infertility in these females. Hence, there was no evidence for a trade-off between longevity and total fecundity, or between longevity and fertility. Elevated reproduction can lead to damage, which may result in reduced lifespan. Our hypothesis is that the activation of autophagy by dietary administration of Torin1 repairs damage caused by elevated reproduction, potentially minimising trade-offs between lifespan and reproductive rate. However, given that autophagy also lowers translation [3], and that we did not directly test here for effects on phospho-S6K, we cannot rule out that the lifespan effects might also be associated with variation in the rate of translation itself.

### Effects of dietary manipulation on lifespan

Extension in lifespan thorough dietary manipulations that activate nutrient sensing and/or repair pathways has been described in many different organisms. Some of this extension of lifespan may arise because of increased protection and repair via removal of damaged macromolecules through increased rates of autophagy [14]. Previous research has tested this idea by activating autophagy through the dietary delivery of spermidine, a natural polyamine which declines in concentration during ageing in humans. Experimental data show that, when added to cells of yeast, flies, worms and human immune cells, spermidine can extend lifespan [2,36,37].

However, spermidine can also be an unreliable activator of autophagy, giving inconsistent results in longevity studies (JSM, TW and TC, unpublished data; [37]). It is possible that spermidine only partially inhibits part of the TOR pathway, i.e. that involving TORC1. Resveratrol is another dietary additive that has been tested in lifespan studies in many species, and which has been proposed to exert its effects at least partly through autophagy. In mice, resveratrol extended the lifespan in obese subjects, but there was no evidence for prolonged life in healthy individuals [38]. Both resveratrol and spermidine therefore seem to have inconsistent effects upon autophagy activation and upon lifespan. Spermidine and resveratrol given in combination may achieve a more consistant induction of autophagy [39]. Further work is needed to assess the relevant mechanisms involved.

As discussed in the introduction, rapamycin has been used widely for extending lifespan, and is proposed to act via the activation of autophagy through inhibition of TORC1. However, it is also reported that although rapamycin can lead to lifespan extension in flies, its effects are not dose dependent [5], suggesting that there are aspects to the underlying mechanism involved, such as for example feedback loops, that remain to be elucidated. Treatment with rapamycin also reveals sexual dimorphism for lifespan extension in flies and mice [5,40].

We chose Torin1 for our studies because of the pressing requirement for additional inhibitors of TOR/activators of autophagy. Our results show that dietary application of Torin1 did activate autophagy. Pyrazolopyrimidines such as PP242, PP30 and Torin1 appear to inhibit both TORC1 and TORC2 with a high degree of selectivity [8,24]. It is believed that although Torin1 inhibits both the TORC pathways it is the more complete inhibition of TORC1 that causes the more reliable activation of autophagy [22,24]. We did observe some variation in the effect size of lifespan extension, it was observed in both experiments and in both sexes and in once-mated and fully reproductive females, yet was inconsistent in the 1μM Torin1 once-mated females across both experiments. This variation was, however, consistent with the apparently greater activation of autophagy in the 1 μM Torin1 fed once-mated females in the first in comparison to the second experiment (S1 Methods, S1 and S2 Figs). The variation in lifespan effects across experiments is important though, because it may suggest that induction of autophagy alone (as occurred across both experiments) may not be sufficient to extend lifespan under the experimental conditions tested.

Once mated males had the longest lifespans in the experiment in which once mated individuals of both sexes were compared. The magnitude or even direction of sex differences in lifespan can vary, according to yeast levels (e.g. [41]) and is also affected by mating status [42–44]. Greater longevity of males over females can occur at low and high nutritional levels [41], and could indicate that the flies in our experiments were relatively underfed, which could contribute to their relatively short lifespans (see also below). Though clearly much more work is needed, our results showing evidence for a simple dose response effect of Torin1 on lifespan via induction of autophagy suggest that Torin1 may provide a potentially useful reagent for ageing studies.

We acknowledge that the lifespans we observed in these assays were typically much shorter than for once mated individuals maintain on full culture media in the absence of live yeast supplementation [5,29] (though for the fully reproductive individuals the lifespans are not out of the typical range for experiments using live yeast [45]). The shorter lifespans were most likely due to a nutritional effect of culturing the flies in these experiments in agar vials in the presence of added live yeast and / or due to exposure to DMSO (in which we dissolved the Torin1). In pilot studies, we found that the agar vial culturing method appeared to deliver more consistent autophagy activation than maintenance on normal food media (Mason, Wileman, Chapman, unpublished data). It also allowed the delivery of Torin1 undiluted by ancillary feeding on other food sources. We did ensure here that fecundity values of females held in the agar vials were similar to those for females held on normal media (see S1 Table). The important point is whether the shorter lifespans in these experiments interacts with the effects of torin1 and there was no evidence for this. For example, even if lifespans were shorter because the flies were accessing less protein in this experimental set up, the observed correlation between longevity under low and normal nutrition (e.g. [46]) supports the idea that our lifespan observations hold. However, future work could usefully search for new TORC inhibitors with better solubility and bioavailability [47].

### Effects of dietary manipulation on fecundity and fertility

Many studies of lifespan extension report trade-offs with fecundity and/or fertility, but this was not found in the results we report here. We observed that fecundity was significantly higher in the longer-lived females fed Torin1. However, the number of fertile eggs produced by these females was lower, resulting in equivalent fertility across Torin1 fed females and controls. Hence there was no evidence of a trade-off between lifespan extension and fertility upon treatment with Torin1. Fecundity dropped after a period of 8–10 days, which could reflect low levels of nutrition in these experiments, as described above. The higher level of egg infertility could also be due to an effect of Torin1 (via the TOR pathway) on cell proliferation [3,8,9], a possibility that would be interesting to test further. Given that rapamycin is an anti-fungal agent, if Torin1 has the same effect, then it is possible that the provision of Torin1 in live yeast paste might have had an inhibitory effect on the yeast itself, and hence possibly also on fecundity. Against this is our observation that fecundity was, at least initially, at a level comparable with that on normal food media, though direct tests of this idea would be useful. The continuously mated females in our experiments appeared to sustain higher egg laying for longer than the once mated flies, which was perhaps unexpected, but was likely due to the tail of the egg laying period overlapping with the beginning of increased death rates. Hence frailty may have dampened down the levels of fecundity. Bjedov *et al*. [5] found a dose-dependent effect of rapamycin treatment on fertility, such that increased rapamycin reduced fertility. However, although lifespan was also extended by rapamycin, the lifespan effect itself was not dose dependent, indicating that the effects of rapamycin on fecundity and lifespan were not closely linked. Further evidence which supported this idea was that the lifespan of sterile mutant females that could not lay eggs was also extended by rapamycin [5].

It has long been believed that reproductive rate and longevity are causally and inversely correlated. A causal relationship is presumed to exist between current reproduction and future survival because limited resources used for reproduction cannot then be used for maintenance of the soma and hence contribute to survival. Risks associated with reproductive activity can lead to bodily injury which may also result in decreased longevity [48]. Our results support the idea that there is no obligate trade-off between reproduction and longevity. This finding fits with an emerging view that lifespan and reproductive rate are intergrated, but may be controlled independently of one another [29]. In our study early fecundity was even enhanced in longer-lived females fed Torin1, although this did not translate to an overall increase in fertility. Our data contribute to the view that survivorship and the timing and amount of reproduction may be controlled by factors other than a simple relationship between longevity and reproduction in the life history of many organisms. Other factors such as male-female interactions may be a more important determinant of female survival than reproductive rate itself.

The enhanced fecundity in females fed Torin1 had no apparent fitness value to the female. It is possible that the elevated egg production traded off against a reduced effiency of fertilisation, or that there were pleiotropic effects of the manipulations on reproduction overall. More work is needed to investigate this effect. Consistent with the idea of pleiotropy is that it has recently been shown that spermidine is essential for mating in yeast as well as for egg fertilization in the nematode *C*. *elegans*. These effects were in both cases found to occur independently from autophagy activation state [49]. It is also unclear why some activators of autophagy, such as rapamycin, can reduce fecundity, but others such as Torin1, do not as we showed here. These two pharmacological agents differ in their relative effects on TORC1 and 2, as described in the introduction, and the lifespan extension by Torin1 (this study), but not rapamycin, appears to be dose-dependent [5]. The exact reason for the fecundity differences remain to be elucidated.

It has been shown that cells exposed to Torin1 become subject to a regulatory feedback loop [23,50]. In experiments employing low doses of Torin1 in an acute treatment of 1 to 2 hours, TORC1 and TORC2 can be inhibited, but this effect is subsequently nullified by an increase in P13K activity at longer time points of 5 hours and over. For this reason it is recommended that does of 250nM at least are used for all long-term exposure experiments, otherwise this regulatory feedback loop negates any effect of the experimental manipulations on autophagy state. Peterson *et al*. [50] found that at a low dose of 50nM Torin1 inhibits both TORC1 and TORC2. However, after acute exposure Akt^S473^ phosphorylation recovered by 48-h despite the fact that S6K1^T389^ remains dephosphorylated [23,50]. The findings suggested that 50nM Torin1 is not sufficient to inhibit all of the TORC2 complexes as suggested above. A higher dose of Torin1 inhibits TORC1 more completely and blocks Akt^S473^ phosphorylation after 48 hours [23]. These results suggest that the choice of Torin1 dose is particularly important in order for predicted and sustained effects on autophagy to be observed. Our dose response experiment was consistent with this idea in that increasing levels of Torin1 in the diet up to 5μM increased autophagy activation, but above this level autophagy activation slightly decreased.

Our overall conclusion is that Torin1 can increase in lifespan in flies. The results contribute to the accumulating evidence that dietary pharmacological manipulation can result in an increase in longevity in whole organisms through the stimulation of autophagy. Further research should be undertaken to determine the role of autophagy in relation to ageing and longevity. It could be particularly informative, for example, to investigate whether a diet containing Torin1 or that is rich in a combination of natural pro-autophagic components can promote health and postpone ageing in humans.

## Supporting information

S1 Methods Western blot analysis for detection of autophagy activation by Torin1 in whole flies. (PDF)

S2 Methods Fecundity of females maintained on live yeast supplemented agar, versus live yeast supplemented SYA medium. (PDF)

S3 Methods Effect of DMSO on once mated female survival. (PDF)

S1 Table Fecundity of once-mated females held in agar vials with yeast droplet. Mean egg production (±SE) over 3 days for once-mated females maintained in agar or SYA medium vials, each containing a droplet of live yeast paste (2mm diameter).
(PDF)

S2 Table Raw survival and fecundity data. Individual-level longevity and fecundity raw data for the experiments described in this study.
(XLS)

S1 Fig Autophagy activation in once-mated females maintained for 5 days on food medium containing 0.5–10 μM Torin1. (A) Normalised Atg8-I/Atg8-II ratios of once-mated single females exposed to DMSO carrier control (black) or 0.5 μM (yellow), 1 μM (orange), 5 μM (red) and 10 μM (green) Torin1 in the diet. Cleavage of Atg8 to Atg8-I and Atg8-II indicates the activation of autophagy and the ratio of the two cleavage products indicates the extent of autophagy activation. (B) Western blot from which the ratios shown in (A) were derived. Atg8-I and Atg8-II ratios of once mated single females were: DMSO control (0.38), 0.5μM (0.44), 1μM (0.58), 5μM (0.60) and 10μM (0.49) Torin1. Both *Atg8-I* and *Atg8-II* were normalised against the control tubulin and the extent of cleavage calculated by determining the ratio between the normalised values of *Atg8-II* and *I*. Spearman’s rank correlation analysis gave rho = 0.7, p < 0.05, suggesting a significant positive relationship between Torin 1 dose and autophagy activation (as measured by Atg8 I/II ratio).
(PDF)

S2 Fig Autophagy activation in control, Torin1 and starved once-mated females and males and continually mated females. (A) Normalised Atg8-I/Atg8-II ratios of once-mated (red) and continually mated (purple) females and once-mated males (blue) held for 5 days on DMSO carrier control food (solid), 1 μM Torin1 (striped) or agar only food (starved, stippled bars). Cleavage of Atg8 to Atg8-I and Atg-II indicates the activation of autophagy and the ratio of the two cleavage products indicates the extent of autophagy activation. (B) Western blot from which the ratios shown in (A) were derived. *Atg8-I/Atg8-II* ratios were: DMSO control: once-mated females = 0.26, fully reproductive females = 0.03, once-mated males = 0.09; 1 μM Torin1: once-mated females = 0.34, fully reproductive females = 0.073, once-mated males = 0.25; starved (agar only): once-mated females = 0.40, fully reproductive females = 0.20, once-mated males = 0.11. There are insufficient data to conduct a rigorous statistical analysis of all individual ratios. However, all ratios are higher for males and females in the Torin1 > control and in the starved > control treatments, indicating that Torin1 and starvation both increase autophagy in both sexes.
(PDF)

S3 Fig Survival of once-mated females on DMSO. Survivorship against time (in days) of once-mated females held on agar vials seeded with 0, 1 or 5μl DMSO yeast droplet treatments.
(PDF)
