# Supplementary material for: Lifespan extension without fertility reduction following dietary addition of the autophagy activator Torin1 in Drosophila melanogaster
Source: PLoS One. 2018 Jan 12;13(1):e0190105. doi: 10.1371/journal.pone.0190105 (PMC5766080; doi:10.1371/journal.pone.0190105)
Supplement: S1 Methods — (PDF) [file pone.0190105.s001.pdf]

Lifespan extension without fertility reduction following dietary addition of the autophagy activator Torin1 in *Drosophila melanogaster*

Janet S. Mason[1], Tom Wileman[2], Tracey Chapman[1]

[1]School of Biological Sciences, University of East Anglia, Norwich Research Park, Norwich, NR4 7TJ, UK

[2]Norwich Medical School, University of East Anglia, Norwich Research Park, Norwich, NR4 7TJ, UK

S1 Methods: Western blot analysis for detection of autophagy activation by Torin1 in whole flies.

Activation of autophagy was determined by detecting cleavage of the *Atg8* gene protein into Atg8-I and Atg8-II subunits, by using Western blotting analysis [1]. The intensity of the separate cleaved bands was recorded and normalized against the intensity of the tubulin control for each lane (as in [1]). Ten flies were sampled from each of the doses used in the dose response experiment for validation of autophagy activation (carrier only (DMSO) control group (Con), 0.5 µM, 1 µM, 5 µM and 10 µM Torin1). The procedure was similar for the second experiment in which the effect of a single dose (1 µM) of Torin1 was used in once-mated females and males and in continually-mated females. Here we also tested for activation of autophagy activation by Torin1 in comparison to by starvation. This was to determine to what extent Torin1 activation of autophagy could recapitulate that induced by 5 days of starvation on agar only food.

Flies were crushed after freezing in liquid nitrogen 5 days after being placed on required dose of Torin1 or agar only food, and homogenised in 100 µl lysis buffer (120 mM NaCl, 50 mM Tris-HCl, 20 mM NaF, 1 mM Benzamidine, 1 mM EDTA, 6 mM EGTA, 15 mM $NA_4P_2O_7$, 1% Nonidet P-40) containing protease inhibitors. Proteins were separated on a 15% SDS-PAGE gel and blotted onto Immobilon FL transfer membrane (Millipore IPFL00010). Primary antibodies were applied overnight at 4°C, anti-*dAtg8- 1*:1000 (kindly supplied by K. Köhler [2]), anti-tubulin1:10000 (abcam ab160) and secondary antibodies, anti-rabbit 1:10000 (LI-COR 926-32211) and anti-rat 1:10000 (LI-COR 926-68076) for 1 hour and 40 minutes. For quantification of Western blot signals, image software from odyssey (LI-COR) was used to calculate the intensity value of the bands on the different doses of Torin1. The same procedures were used for the once-mated, continually-mated females and the once-mated males. The fluorescent inensity of both Atg8-I and Atg8-II were then normalized against the tubulin control and the extent of cleavage calculated by determining the ratio between the normalized values of Atg8-I and II.

Dose response of Torin1 on autophagy activation in once-mated females fed 0.5-10 µm Torin 1.

The Western blotting assays described above confirmed that the administration of Torin1 in the diet activated autophagy above the control diet level at all doses of Torin1 in the once-mated females from the first experiment (S1 Fig). Increasing doses of Torin1 successively increased the level of autophagy activation up to 5 µm Torin1. Autophagy activation then slightly decreased at the highest level of 10 µm Torin1.

Dose response of Torin1 on autophagy activation in once-mated males and females and continually mated females fed 1 µM Torin 1.

Dietary administration of Torin1 also activated autophagy above the control diet level in once-mated females and males and in continually-mated females all fed 1 µM Torin1 in the second experiment (S2 Fig). In once-mated and continually mated females, autophagy activation following 5 days on 1 µM Torin1 diets was higher than in individuals maintained on the control diet, but was not as high as for the starvation diet females maintained on agar only food. In once-mated males, autophagy activation on the 1 µM Torin diet exceeded that of males maintained on the starvation agar only diet. Together, the results show that autophagy was indeed activated by Torin1 in the diet in both experiments.

References

1. Klionsky DJ, Abeliovich H, Agostinis P, Agrawal DK, Aliev G, et al. Guidelines for the use and interpretation of assays for monitoring autophagy in higher eukaryotes. Autophagy 2008;4:151-175.
2. Barth JMI, Szabad J, Hafen E, Köhler K. Autophagy in Drosophila ovaries is induced by starvation and is required for oogenesis. Cell Death Differ. 2011;18:915-924.
