# Supplementary material for: Lifespan extension without fertility reduction following dietary addition of the autophagy activator Torin1 in Drosophila melanogaster
Source: PLoS One. 2018 Jan 12;13(1):e0190105. doi: 10.1371/journal.pone.0190105 (PMC5766080; doi:10.1371/journal.pone.0190105)
Supplement: S2 Methods — (PDF) [file pone.0190105.s002.pdf]

Lifespan extension without fertility reduction following dietary addition of the autophagy activator Torin1 in *Drosophila melanogaster*

Janet S. Mason[1], Tom Wileman[2], Tracey Chapman[1]

[1]School of Biological Sciences, University of East Anglia, Norwich Research Park, Norwich, NR4 7TJ, UK

[2]Norwich Medical School, University of East Anglia, Norwich Research Park, Norwich, NR4 7TJ, UK

S2 Methods: Fecundity of females maintained on live yeast supplemented agar, versus live yeast supplemented SYA medium

We tested whether, in this experimental set up, females maintained in agar vials supplemented with live yeast paste had comparable fecundity with those held on normal SYA food medium supplemented with live yeast in the same manner (n=30 per treatment group). To ensure that both sets of females were eating the live yeast, we also added blue food dye (50mg dye (E133 Brilliant Blue)/100ml) and then added a droplet the resulting yeast paste solution to agar or SYA medium vials. Once-mated wild type females were maintained in these vials for 3 days and transferred to new food every day. Female fecundity was recorded daily. Females were examined at the end of this period and it was found that the intensity of blue colour in their abdomens was comparable (data not shown), suggesting that both groups were eating the live yeast. There were no significant differences in fecundity between the two groups on any of the 3 days tested ($F_{1,169}$=1.471, p=0.227, S1 Table) or across all 3 days combined (p=0.227). These data suggest that the nutritional conditions under which the females were held in the main experiments, i.e. in agar vials with yeast droplets, allowed females to express fecundity comparable with that observed using standard culturing vials.
