# Supplementary material for: Lifespan extension without fertility reduction following dietary addition of the autophagy activator Torin1 in Drosophila melanogaster
Source: PLoS One. 2018 Jan 12;13(1):e0190105. doi: 10.1371/journal.pone.0190105 (PMC5766080; doi:10.1371/journal.pone.0190105)
Supplement: S3 Methods — (PDF) [file pone.0190105.s003.pdf]

Lifespan extension without fertility reduction following dietary addition of the autophagy activator Torin1 in *Drosophila melanogaster*

Janet S. Mason[1], Tom Wileman[2], Tracey Chapman[1]

[1]School of Biological Sciences, University of East Anglia, Norwich Research Park, Norwich, NR4 7TJ, UK

[2]Norwich Medical School, University of East Anglia, Norwich Research Park, Norwich, NR4 7TJ, UK

S3 Methods: Effect of DMSO on once-mated female survival

We tested the effect of the DMSO carrier per se on once mated female lifespan, to ascertain whether there was evidence for any toxic effect of DMSO on lifespan at the dose applied in the main experiments (in which 5 µL DMSO was added to 10 mL distilled water to which 6 g live yeast granules were added). Once-mated females were reared as before, anaesthetized using CO_2 and randomly assigned to one of 3 treatment groups (n=30 per treatment) 24 hours following eclosion. Each vial contained an agar plug to which a droplet of yeast paste (2mm diameter) was added, from the 0, 1 or 5µl of DMSO treatments (the 5µ L treatment being the same as used in the main experiments). Flies were transferred between vials without using anaesthesia twice a week until death and longevity was scored daily. There were no significant differences in once mated female survivorship in the 0, 1 and 5 µL treatments (mean survival 36.8, 40.9, 33.7, respectively; Log Rank tests, all pairwise comparisons $p > 0.05$; S3 Fig).
