# Supplementary material for: Lifespan extension without fertility reduction following dietary addition of the autophagy activator Torin1 in Drosophila melanogaster
Source: PLoS One. 2018 Jan 12;13(1):e0190105. doi: 10.1371/journal.pone.0190105 (PMC5766080; doi:10.1371/journal.pone.0190105)
Supplement: S1 Table — Mean egg production (±SE) over 3 days for once-mated females maintained in agar or SYA medium vials, each containing a droplet of live yeast paste (2mm diameter). (PDF) [file pone.0190105.s004.pdf]

S1 Table. Fecundity of once-mated females held in agar vials with yeast droplet. Mean egg production (±SE) over 3 days for once-mated females maintained in agar or SYA medium vials, each containing a droplet of live yeast paste (2mm diameter).

	Mean egg production			
Treatment	Day 1 (±SE)	Day 2 (±SE)	Day 3 (±SE)	All 3 days combined
Agar vials + yeast droplet	44 (2.2)	55 (1.6)	66 (3.0)	55 (1.6)
SYA vials + yeast droplet	39 (2.0)	60 (2.2)	77 (4.1)	59 (2.3)
