# Supplementary material for: Lifespan extension without fertility reduction following dietary addition of the autophagy activator Torin1 in Drosophila melanogaster
Source: PLoS One. 2018 Jan 12;13(1):e0190105. doi: 10.1371/journal.pone.0190105 (PMC5766080; doi:10.1371/journal.pone.0190105)
Supplement: S1 Fig — (A) Normalised Atg8-I/Atg8-II ratios of once-mated single females exposed to DMSO carrier control (black) or 0.5 μM (yellow), 1 μM (orange), 5 μM (red) and 10 μM (green) Torin1 in the diet. Cleavage of Atg8 to Atg8-I and Atg8-II indicates the activation of autophagy and the ratio of the two cleavage products indicates the extent of autophagy activation. (B) Western blot from which the ratios shown in (A) were derived. Atg8-I and Atg8-II ratios of once mated single females were: DMSO control (0.38), 0.5μM (0.44), 1μM (0.58), 5μM (0.60) and 10μM (0.49) Torin1. Both Atg8-I and Atg8-II were normalised against the control tubulin and the extent of cleavage calculated by determining the ratio between the normalised values of Atg8-II and I. Spearman’s rank correlation analysis gave rho = 0.7, p < 0.05, suggesting a significant positive relationship between Torin 1 dose and autophagy activation (as measured by Atg8 I/II ratio). (PDF) [file pone.0190105.s006.pdf]

S1 Fig

A

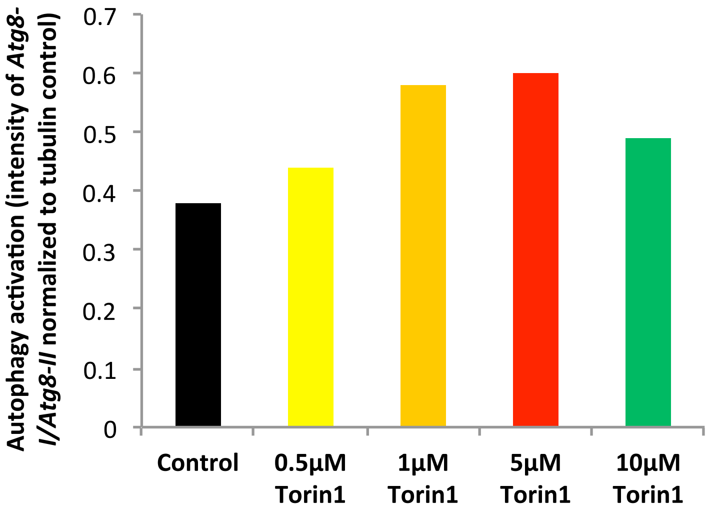

B

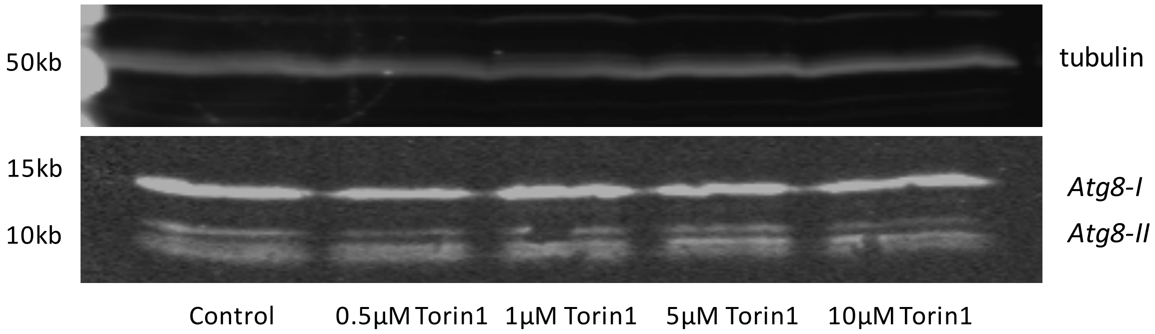

S1 Fig. Autophagy activation in once-mated females maintained for 5 days on food medium containing 0.5-10 µM Torin1. (A) Normalised Atg8-I/Atg8-II ratios of once-mated single females exposed to DMSO carrier control (black) or 0.5 µM (yellow), 1 µM (orange), 5 µM (red) and 10 µM (green) Torin1 in the diet. Cleavage of Atg8 to Atg8-I and Atg8-II indicates the activation of autophagy and the ratio of the two cleavage products indicates the extent of autophagy activation. (B) Western blot from which the ratios shown in (A) were derived. Atg8-I and Atg8-II ratios of once mated single females were: DMSO control (0.38), 0.5µM (0.44), 1µM (0.58), 5µM (0.60) and 10µM (0.49) Torin1. Both *Atg8-I* and *Atg8-II* were normalised against the control tubulin and the extent of cleavage calculated by determining the ratio between the normalised values of *Atg8-II* and *I*. Spearman's rank correlation analysis gave rho = 0.7, $p < 0.05$, suggesting a significant positive relationship between Torin 1 dose and autophagy activation (as measured by Atg8 I/II ratio).
