# Supplementary material for: Lifespan extension without fertility reduction following dietary addition of the autophagy activator Torin1 in Drosophila melanogaster
Source: PLoS One. 2018 Jan 12;13(1):e0190105. doi: 10.1371/journal.pone.0190105 (PMC5766080; doi:10.1371/journal.pone.0190105)
Supplement: S2 Fig — (A) Normalised Atg8-I/Atg8-II ratios of once-mated (red) and continually mated (purple) females and once-mated males (blue) held for 5 days on DMSO carrier control food (solid), 1 μM Torin1 (striped) or agar only food (starved, stippled bars). Cleavage of Atg8 to Atg8-I and Atg-II indicates the activation of autophagy and the ratio of the two cleavage products indicates the extent of autophagy activation. (B) Western blot from which the ratios shown in (A) were derived. Atg8-I/Atg8-II ratios were: DMSO control: once-mated females = 0.26, fully reproductive females = 0.03, once-mated males = 0.09; 1 μM Torin1: once-mated females = 0.34, fully reproductive females = 0.073, once-mated males = 0.25; starved (agar only): once-mated females = 0.40, fully reproductive females = 0.20, once-mated males = 0.11. There are insufficient data to conduct a rigorous statistical analysis of all individual ratios. However, all ratios are higher for males and females in the Torin1 > control and in the starved > control treatments, indicating that Torin1 and starvation both increase autophagy in both sexes. (PDF) [file pone.0190105.s007.pdf]

S2 Fig

A

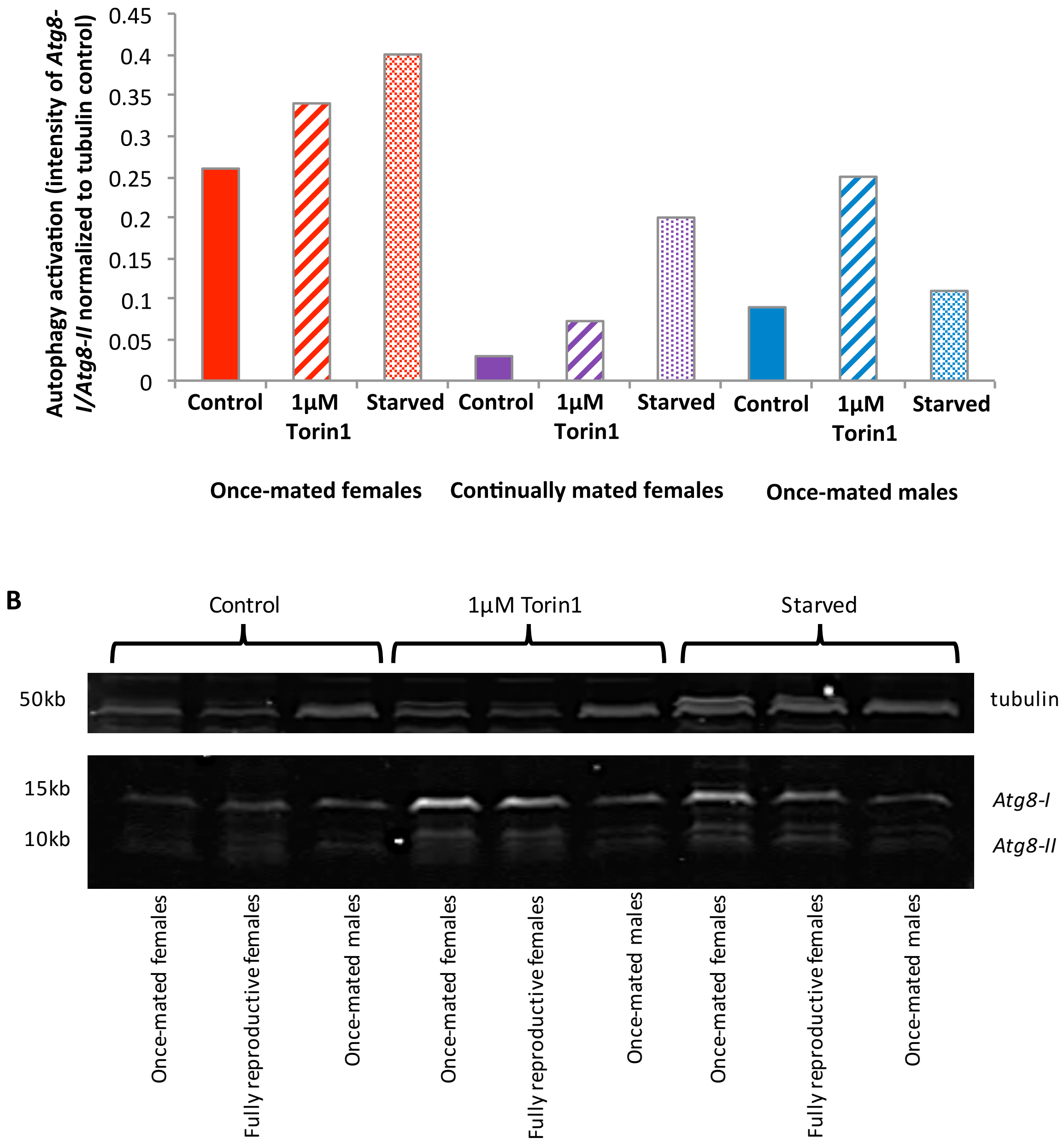

S2 Fig. Autophagy activation in control, Torin1 and starved once-mated females and males and continually mated females. (A) Normalised Atg8-I/Atg8-II ratios of once-mated (red) and continually mated (purple) females and once-mated males (blue) held for 5 days on DMSO carrier control food (solid), 1 µM Torin1 (striped) or agar only food (starved, stippled bars). Cleavage of Atg8 to Atg8-I and Atg-II indicates the activation of autophagy and the ratio of the two cleavage products indicates the extent of autophagy activation. (B) Western blot from which the ratios shown in (A) were derived. *Atg8-I/Atg8-II* ratios were: DMSO control: once-mated females =0.26, fully reproductive females =0.03, once-mated males =0.09; 1 µM Torin1: once-mated females = 0.34, fully reproductive females = 0.073, once-mated males = 0.25; starved (agar only): once-mated females = 0.40, fully reproductive females = 0.20, once-mated males = 0.11. There are insufficient data to conduct a rigorous statistical analysis of all individual ratios. However, all ratios are higher for males and females in the Torin1 > control and in the starved > control treatments, indicating that Torin1 and starvation both increase autophagy in both sexes.
