# Supplementary material for: Lifespan extension without fertility reduction following dietary addition of the autophagy activator Torin1 in Drosophila melanogaster
Source: PLoS One. 2018 Jan 12;13(1):e0190105. doi: 10.1371/journal.pone.0190105 (PMC5766080; doi:10.1371/journal.pone.0190105)
Supplement: S3 Fig — Survivorship against time (in days) of once-mated females held on agar vials seeded with 0, 1 or 5μl DMSO yeast droplet treatments. (PDF) [file pone.0190105.s008.pdf]

S3 Fig

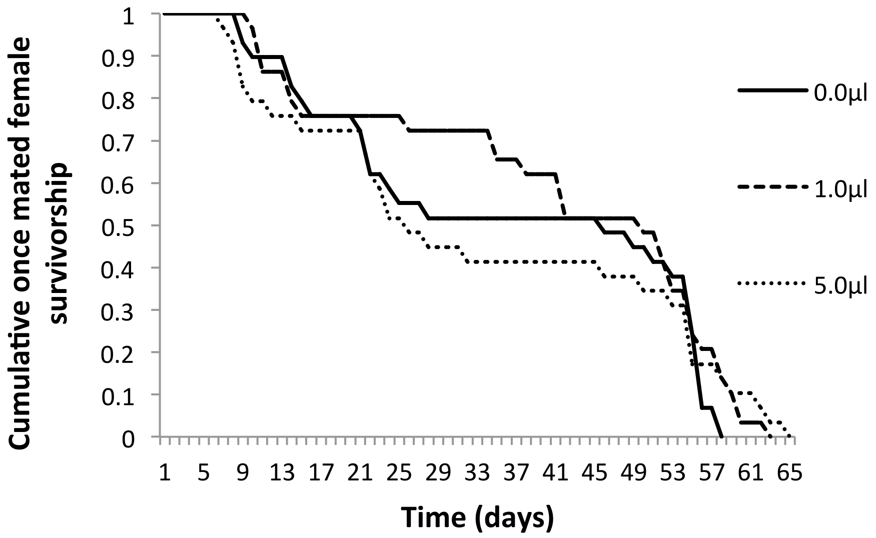

S3 Fig. Survival of once-mated females on DMSO. Survivorship against time (in days) of once-mated females held on agar vials seeded with 0, 1 or 5µl DMSO yeast droplet treatments.
